# The what and why of interprofessional collaboration according to first-year health professions students: a thematic analysis of written reflections

**DOI:** 10.1186/s12909-025-08375-1

**Published:** 2025-12-01

**Authors:** Maria Kvarnström, Samuel Edelbring, Annika Lindh Falk

**Affiliations:** 1 Department of Health, Medicine and Caring Sciences, Linköping University, Linköping, Sweden; 2 School of Education, Culture and Communication, Mälardalen University, Västerås, Sweden; 3 School of Health Sciences, Örebro University, Örebro, Sweden

**Keywords:** Health professions education, Interprofessional education, Interprofessional learning, Interprofessional competence, Medical education, Thematic analysis, Reflective writing

## Abstract

**Background:**

Interprofessional education (IPE) is highly recommended in health professions education, but there is still an ongoing discussion regarding when to introduce interprofessional educational activities. The development of students’ professional and interprofessional competence during education is essential for fostering effective collaboration in future healthcare settings. This study explored how health professions students, in connection with their first IPE experience, reflect upon and express interprofessional competencies in relation to future collaborative work.

**Methods:**

A qualitative design was employed using a reflexive thematic analysis based on first-year students’ reflective writings, written during the first IPE module in a mandatory curriculum.

**Results:**

Four themes were identified, representing crucial components for effective interprofessional collaboration. These themes are communication as a fundamental factor, professional knowledge as a common foundation, establishing trust within a workgroup, and, finally, navigating hierarchies in the evolving healthcare landscape. The students elaborated on these themes in relation to the context of collaborative work in professional teams, and emphasised the importance of the quality of care and patients’ treatment outcomes.

**Conclusions:**

This study contributes to the discussion of the value of introducing IPE at an early stage of health professions education. The findings demonstrated that students can elaborate on interprofessional collaboration and patient safety in this early IPE intervention using brief reflective writing as part of an IPE module. With a clear focus on future professional work, the students identified interprofessional competencies as a part of their learning process.

## Background

There is a long tradition of implementing interprofessional education (IPE) in undergraduate health programmes. Learning activities and settings vary, e.g., between a theoretical workshop lasting for a few hours [1, 2], simulation sessions [3–5], interprofessional activities during clinical placement [6–10] and interprofessional training wards [11–13]. Most IPE activities are implemented as standalone activities, while others form a part of an IPE curriculum, integrated into the profession-specific curriculum as a step-by-step learning process [14]. The integrated approach to IPE supports the development of interprofessional competence over time [14–16].

During the long history of IPE curricular design, the question of when IPE should be introduced has surfaced without reaching a conclusive answer (e.g. [17]). A review of early IPE implementation suggested that a limited understanding of health professionals’ roles may constrain early IPE activities, and that IPE might be more effective when learners have developed their professional identity and gained patient experiences [18]. On the other hand, several researchers have recommended the introduction of IPE early on, with the overall goal of preventing barriers between professionals and countering negative attitudes and stereotypes towards others [19–21]. Previous research regarding early IPE activities has focused mostly on students’ attitudes, with an indication of positive changes in attitudes and perceptions with respect to other professions and knowledge [20, 22–24].

Researchers have also emphasised that, for students engaging in early IPE, the primary focus is on understanding the roles of other healthcare professionals and recognising the importance of effective communication to ensure patient safety and support patient-centred care [25]. These competencies play vital roles in successful collaboration, and are highlighted as core interprofessional competencies across various established frameworks, such as the Interprofessional Education Collaborative (IPEC) framework [26] and the CIHC Competency Framework for Advancing Collaboration [27]. The particular knowledge and skills that undergraduate students should reach in IPE should be explicitly articulated [22]. Regardless of contextual and cultural variations, these frameworks converge on competencies such as role and responsibility, teamwork, interprofessional communication, leadership, ethics and values. To nurture these key interprofessional core competencies, educational strategies must also go beyond theoretical learning. A more ambitious strategy is to combine educational activities with reflective writing, which serves as a powerful tool in IPE [5, 28]. By engaging in written reflections, students can process their experiences, build confidence and deepen their understanding of interprofessional competences. Furthermore, reflection is a valuable teaching strategy for evaluating students’ comprehension of the complex concepts introduced in IPE [29].

While the literature provides insights into various types of IPE design and how students’ attitudes towards interprofessional collaboration evolve during their education, there is still a limited understanding of what students actually learn about interprofessional collaboration during the early stages of IPE exposure.

The research context for this study is the Faculty of Medicine and Health Sciences at Linköping University, a site with long-term experience in IPE [30, 31]. The current comprehensive curriculum for IPE, implemented in 2016, consists of three mandatory modules in progression, worth a total of 12 European Credit Transfer System (ECTS) credits [14, 32], see Table 1.

**Table 1:** Overview of the interprofessional modules at the faculty of medicine and health sciences at Linköping university

Module	Professionalism in Healthcare	Quality Improvement and Learning in Practice	Professional Perspectives in Collaboration (IPTW)
**Students**	BMLS, M, N, OT, PT, SLP	BMLS, M, N, OT, PT, SLP	BMLS, M, N, OT, PT
**Setting**	Semester 1 (SLP: 2)	Semester 5 (M: 11)	Semester 6 (M: 9)
**Credits (hours)**	6 ECTS (160 h)	3 ECTS (80 h)	3 ECTS (80 h)
**Design/outline**	PBL tutorial groups, seminars, group assignment	PBL tutorial groups, seminars, field study, group assignment	Interprofessional student teams at a clinical ward or a primary healthcare unit

In addition to IPE, all programmes incorporate a problem-based learning (PBL) approach, including tutorial groups, which enables collaboration and aligns well with IPE [33–35]. The shared IPE curriculum is designed to establish a common foundation for future collaboration, covering areas such as legislation, the roles and responsibilities of professions within healthcare and social services, concepts of health, ethical principles, quality improvement, and evidence-based practice related to professional and interprofessional competence development. The IPE activities in the IPE curriculum are both theoretical and practical and have been intentionally designed around the core competencies for interprofessional collaborative practice [32] established by the IPEC [26] framework. These competency domains serve as the guiding framework for constructing the intended learning outcomes. The overall curricular design in this study context, with an IPE module during the first year of study, provides a unique opportunity to explore first-year students’ novel perspectives on interprofessional collaboration, which can provide educators with important directions for shaping future IPE activities later in their education.

The specific aim of this study is to explore how health professions students, in connection with their first IPE experience, reflect upon and express interprofessional competencies in relation to future collaborative work.

## Methods

The study employed a qualitative design with thematic analysis of first-year health professions students’ reflective writings, written during the first IPE module in the curriculum.

The participants in the first IPE module (6 credits) were students from biomedical laboratory science (BMLS), nursing (N), medicine (M), occupational therapy (OT), physiotherapy (PT), and speech and language pathology (SLP) programmes (Table 1). The IPE module is included as one of the learning activities in first-year courses. After seven weeks in their profession-specific programme, the students are introduced to the first IPE module. The content of the IPE module focused primarily on the shared theoretical foundations and values of the various professions, which form part of the prerequisites for future collaborative practice. The key subject areas included theories of health, ethics and the concept of becoming a profession. Learning activities included PBL tutorial groups, seminars, a group assignment and an individual written reflection at the end of the module. The individual reflection on the topic of interprofessional collaboration of up to 400 words formed the data for the study. The task required the students to consider the competencies they had developed during this module, as well as the competencies they needed to develop further in order to collaborate with other professions. The assignment was prompted using questions such as “What competencies/knowledge/skills do you think you need to be able to work in a team with other professions?” and “What factors do you see that can facilitate or hinder interprofessional collaboration?”.

### Participants

All students (*n* = 788) in the autumn semester of 2018 and the spring semester of 2019 were invited via their learning platform to share their reflection assignments. All reflections from consenting students (*n* = 323) were anonymised and included (Table 2).

**Table 2:** Participating students

Education programme	Total number	Included
Occupational Therapy	73	36 (49%)
Biomedical Laboratory Science	37	14 (37%)
Physiotherapy	90	38 (42%)
Speech and Language Therapy	31	13 (42%)
Medicine	260	113 (43%)
Nursing	297	109 (36%)
Total	788	323 (41%)

### Analysis

The data were analysed using reflexive thematic analysis [36]. The different IPE experiences of the research team were consciously and constructively used in the analysis. The authors benefit from diversity in professional perspectives, but share a social constructivistic epistemological position and experience of IPE teaching and research. The social constructivistic epistemological position views knowledge as socially constructed, and hence the researchers are aware of the influence of their own perspectives in the analytical process and acknowledge themselves as co-creators of the findings. This epistemological position is well aligned with the reflective approach to thematic analysis [36]. None of the researchers had teaching or assessment roles in the IPE module.

In the initial phase, two of the researchers (ALF and MK) read all the reflections and one researcher (SE) read a selection of the reflections to gain familiarity with the content. Following this, the researchers individually coded each reflection document in relation to the study aim. After collaborative calibration, the initial codes were refined into a code book using the NVIVO software [37]. In a deeper familiarisation process, and as a step towards identifying themes, a theory-driven approach [38] based on the four interprofessional competencies described in the IPEC framework [26] was used to form temporary categories. Within each category, an explorative inductive approach was then applied, which became the primary analytical approach to form thematic findings. As themes were identified the categories were dissolved, making themes connect to codes and the primary data.

## Results

Based on the thematic analysis of the students’ written reflections, we identified four themes representing crucial components for effective interprofessional collaboration: Communication as a fundamental component, Professional knowledge as a common foundation, Establishing trust within a workgroup, and Navigating hierarchies in the evolving healthcare landscape. With an emphasis on the first theme, they are not hierarchically ordered. These themes recurred in relation to the context of collaborative work in professional teams, and emphasised the importance of the quality of care and patients’ treatment outcomes. Illustrative quotations have been selected to represent the participating education programmes.

### Communication as a fundamental component

Communication was recognised as crucial component for successful collaboration. Communication encompasses not only sharing one’s expertise and the ability to speak up, but also the ability to listen actively to others and ensure that team members can express their opinions, as well as the ability to give and receive constructive feedback.

Communication was emphasised as being fundamental for interprofessional collaboration and was often noted as the most important skill as healthcare professionals to ensure good quality care for patients.

“I believe that communication is the most important thing we have learned, and it is communication that we always return to if something goes wrong or does not match. If you cannot communicate correctly, you will not get anywhere in healthcare, and many things can go wrong due to misunderstandings and other mishaps. Communication is important with both patients and other healthcare staff.” (Student 56)

“For functional interprofessional teams in healthcare, the different professionals must communicate with each other and with their patient in a good way. Communication is extremely important for patients to receive the care they need by eliminating misunderstandings and misjudgements. Additionally, effective communication is crucial for ensuring that patients are well understood, safe and well-treated. In summary, communication in healthcare plays a central and important role in good, safe and effective care.” (Student 217)

“In healthcare, communication plays a significant role, and effective communication results in effective care. If there is good communication between healthcare providers with different competencies and responsibilities, it contributes to clarity and structure, misunderstandings are avoided, and conflicts are prevented. From a patient perspective, the patient avoids being caught in the middle because relevant information is not conveyed between different healthcare providers involved in the care, while the patient is met by the relevant profession and competence.” (Student 123)

Suboptimal communication was considered a major barrier to effective interprofessional collaboration and high-quality patientcare. How one expresses oneself with others, as well as the ability and willingness to listen to each other, were mentioned as factors that can hinder good collaboration. An awareness of different types of professional jargon was highlighted as important by some of the students, who described the necessity of communication in a way that is understandable to themselves (both colleagues and patients) to reduce misunderstandings.

“Without an open dialogue and an open conversational climate, this does not work. It is essential to listen, ask questions and show respect to facilitate patient care.” (Student 138).

“Constantly maintaining an open dialogue with team members helps ensure that no mistakes occur which could negatively impact the patient.” (Student 267).

“I believe that attentiveness is a crucial quality in team-based work, to ensure everyone’s voices are heard and to give a chance to express one’s thoughts in the discussion.” (Student 261).

“Since each profession uses a specific language, it is important to be aware of how one expresses oneself between professions so that everyone understands, and no misunderstandings occur.” (Student 36).

Finally, the students mentioned a psychosocial and relational climate that encouraged everyone in the team to speak out and take a humble attitude towards the expertise of others.

“Must be open to people’s differences, and something I consider very valuable, and want to work more on, is openness and willingness to learn from others and better understand them, instead of only relying on one’s own values and opinions.” (Student 179).

“Humility is crucial because no single profession has all the answers. A continuous exchange of information and knowledge between professions is essential for effective care and a positive work environment.” (Student 150).

“Not everyone may dare to make their voice heard, and valuable knowledge from each other’s areas of expertise and important improvement efforts may be lost.” (Student 117).

### Professional knowledge as a common foundation

The students noted that despite their different and unique future professional expertise, they share many commonalities, which it is important to be aware of. They highlighted the importance of understanding the competencies of other professionals while also stressing the need to have a solid foundation rooted in one’s own unique professional skills.

The students identified common areas of knowledge, including laws, policies, ethics and collective responsibility for patient safety. Many students felt empowered by recognising the similarities in their knowledge. They believed that they had gained a deeper understanding of their shared values.

“It is good that all students share the same values regarding the view of humanity, including that everyone should be treated with respect, even if it is expressed in different ways.” (Student 276).

“I think it is good that we have seen and been able to discuss things that will affect us all, to notice a greater community in our professions than I have seen and perhaps understood before.” (Student 170).

“One of the best aspects of teamwork is that we all view the patient from different perspectives. Together, we gain various insights and a wealth of knowledge about a specific area, allowing us to address a patient’s concerns collaboratively and truly help the patient from multiple angles through cooperation.” (Student 211).

In addition to common areas, the differences also contributed to the quality of care through collaboration. For this purpose, the students emphasised the importance of understanding the roles and capabilities of other professions. This understanding helps guide patients to appropriate professionals and allows for mutual benefit in their own work. Recognising the boundaries between one’s responsibilities and those of colleagues was described as crucial, as it allows the patient’s needs to be viewed from different perspectives, tasks to be distributed effectively, and professionals to be adaptable when encountering team challenges to achieve the best outcomes.

“Understanding what the other professions do and how everything is connected is, I think, the foundation for interprofessional learning and collaboration.” (Student 18).

“I think it is important to have knowledge about the other professions in your team if you encounter a patient who needs help from another profession, as it is more efficient if I know who they need to go to.” (Student 23).

“I need to be able to view healthcare from the perspectives of other professions to understand and accept their ways of working, their priorities in healthcare and their opinions on the patient’s capabilities, such as whether a patient is ready to go home and live independently.” (Student 28).

“To be strong in my work, even when it is no longer under my responsibility, it is essential to know who should take over the patient and to have good cooperation with them so that the patient feels safe and secure.” (Student 193).

Many students noted that effective collaboration requires a strong understanding of one’s own profession, not only to act professionally and contribute one’s knowledge but also to ensure that patients receive the highest quality of care. Some of the students highlighted the significant responsibility that professionals have to meet societal expectations.

“Good interprofessional collaboration begins with a solid understanding of one’s own profession. This foundational knowledge enables one to contribute effectively to the interprofessional team, meeting the expectations of the team, the patient and society.” (Student 36).

“I need to feel confident in my knowledge and profession. If one feels confident in your competence, I believe you can contribute better to the team, which increases the chances of providing the best possible care.” (Student 153).

### Establishing trust within a workgroup

The students described the importance of being aware of prejudices, emphasising that they need to identify and reflect on prejudices in their future profession and consider how these prejudices impact collaboration with others. Several students noted that they recognised preconceived notions about various professions, highlighting that these biases could impede effective collaboration.

“I gained increased knowledge of several different prejudices in healthcare during this IPE module. This has been a good lesson for the future, not getting involved in stigmatising vulnerable groups. The new knowledge has given me an even stronger perspective on how important it is to have equal care, on the basis of various ethical principles but also in relation to the Health and Medical Services Act.” (Student 39).

“I know that I need to work with prejudices that I have myself, and I try to do that by being curious, questioning and seeking information about them in order to get rid of them.” (Student 48).

The students emphasised the importance of building trust within established and ad hoc working groups. They noted that this trust is crucial not only during training in university group works, but also in their future healthcare careers. They highlighted the need to respect and value each other’s skills, and to create opportunities to set common goals. Additionally, they stressed the importance of a safe environment where everyone feels comfortable.

“There is a need for an open climate where no profession looks down on anyone else but instead has an attitude that everyone’s contribution is important to achieve the most optimal care possible.” (Student 22).

“The personal qualities and skills needed to work effectively in interprofessional teams are deeply rooted in respect. Understanding the significance of your colleagues’ contributions to patient care and realising that everyone is equally important in different ways fosters greater respect and understanding of their actions.” (Student 159).

“In our professions, being alone is not strong; rather, we need each other’s help, support and knowledge to offer the best possible care for our patients.” (Student 212).

### Navigating hierarchies in the evolving healthcare landscape

The issue of hierarchies in healthcare was a recurring theme in the students’ reflections. They described scenarios where physicians are perceived as being at the top, with other professions ranked below—a perception that can be developed early on during undergraduate education. The students believed that these hierarchical patterns, which have historically characterised healthcare, can hinder effective collaboration. They emphasised the need to move away from these outdated structures and instead view each other as valuable resources in a modern healthcare system.

“I believe that attitude is very important for effective interprofessional collaboration. If a person enters with the mindset that they are better than others or does not care about them, I am convinced that it will lead to a poor working environment.” (Student 48).

“I see hierarchy as a factor that can hinder interprofessional teamwork. When I think of a team, it means that everyone has an equal place in the group, collaborating and respecting each other. I do not believe teamwork with hierarchy will work, whether it is one person or one profession trying to be the best. In teamwork, no one person should be the best; instead, you should collaborate and be the best group together.” (Student 313).

“If the organisation does not function well, hierarchies can easily arise, creating unhealthy norms and a silo mentality. Collaboration in such a climate becomes poor, resulting in lower quality of care and negatively affecting the patient.” (Student 320).

The changing healthcare landscape, for example a growing elderly population with comorbidities increasing the need for interprofessional collaboration, was emphasised by some students.

“As people live longer, healthcare will become increasingly burdened. Unfortunately, the healthcare system cannot meet the growing demand for care, making it even more important to work in teams where everyone’s knowledge is used as effectively as possible.” (Student 273).

“In the future, I believe this will be particularly beneficial, as many healthcare cases involve various providers from different professions. Instead of working separately and duplicating unnecessary tasks, it is much more efficient to collaborate and arrive at common solutions to benefit the health of our future patients.” (Student 283).

“Better collaboration streamlines the care chain and improves the quality of healthcare, which is a way to save both resources and staff, reduce patient suffering and shorten waiting times.” (Student 12).

## Discussion

This study is based on an analysis of first-year students’ written reflections following an IPE activity. The findings reveal that, despite being in the early stages of their education, the students demonstrate a notable awareness of interprofessional competencies and their relevance for effective collaboration and patient safety. This awareness was evident across all four themes. Their reflections indicate a mature understanding of the value of interprofessional collaboration—not only in fostering respectful teamwork, but also in addressing the increasing complexity and demands in future healthcare.

The students’ reflections on the importance of understanding and acknowledging patients’ needs and voices constitute a significant and somewhat unexpected finding in this study. While first-year students are usually in the initial stages of professional socialisation into their future roles—a process that is itself important—this study demonstrates that early, well-designed interprofessional education can shift the focus beyond their own professional boundaries. In doing so, the patient emerges as a shared point of reference, highlighting the potential of interprofessional education to foster a patient-centred mindset.

Several students emphasised that developing effective team communication is a critical competency during their education, as it plays an important role in preventing errors in judgement and ensuring that patients receive safe and appropriate care. Misunderstandings among professionals due to poor communication are a widespread factor among various healthcare settings that affects patient safety [39]; therefore, it is important to start becoming aware of the negative impact of misunderstandings. Our findings are consistent with those of Kemp and Brewer [25], who highlighted students’ awareness of the importance of communication skills in enhancing patient safety. The structure of this IPE module in an interprofessional PBL tutorial group setting encourages students to develop their communication skills across professional boundaries. In these learning activities they are required not only to articulate and explain their own professional knowledge, but also to actively listen and engage with the perspectives of peers from other professional programmes. The increased awareness of interprofessional communication is consistent with findings from Törnqvist et al. [40], who emphasise that as students engage collaboratively in PBL tutorial groups, they connect professional knowledge in ways that reflect both shared understanding and profession-specific nuances.

By recognising the value of both unique and shared professional knowledge, some students reflected on the boundaries between different areas of expertise for distributing tasks, adapting to interprofessional collaboration and learning from each other in such situations. These findings are in line with those of Jentoft [41], who claims that “students’ interprofessional abilities are strengthened when different professional perspectives are encountered in situations that require collaboration and negotiation”.

This finding can also be considered in relation to the definition of IPE as a process in which “students from two or more professions learn about, from, and with each other to enable effective collaboration and improve health outcomes” [42, 43]. While the initial part of this definition is often emphasised in IPE design in general, the latter component—which highlights the ultimate goal of enhancing patient care and health outcomes—is less frequently emphasised [43]. This study, however, brings this outcome-oriented dimension to the fore, illustrating that even students at the beginning of their education can recognise and express the centrality of the patient and the transformative potential of IPE in fostering patient-centred, collaborative care. The need to ensure all aspects of the definition, including improved patient outcomes, has recently become crucial for advancing interprofessional science [43].

Finally, the students reflected on organisational aspects of the healthcare system and the risk that hierarchies can hinder interprofessional collaboration. Hierarchy can have a negative impact on several factors, from overall communication and relationships in and between organisations to creating a power imbalance between professions and reducing team engagement [44], and the fact that this awareness emerges already in the early stages of their education is noteworthy. Moreover, the students demonstrated an understanding of how interprofessional collaboration contributes to patient safety, the need for future care and system efficiency. This insight of contributions from different professional competencies to holistic patient care is implicit in each professional education and becomes explicit when students from different professions are brought together in IPE [5].

These findings underscore the value of integrating IPE into health professions education at an early stage, not only to build foundational interprofessional competencies but also to shape professional identities grounded in mutual respect, shared responsibility and a commitment to collaborative practice in increasingly complex healthcare environments.

It is somewhat notable that, despite lack of concrete team and substantial clinical practice experience, the students demonstrated awareness of consequences of hierarchies and power dynamics for the holistic view of patient benefit. The development of this awareness might be connected to the format of the module with students’ exposure to these topics in PBL groups supporting sharing and active reflecting on experiences from each other’s perspectives and emerging professional identities.

The value of the early introduction of IPE is in line with observations from other studies [23, 24], and is explained by the fact that students may be more enthusiastic about learning together early in their education [20]. This enthusiasm is something to benefit from when designing IPE activities. Establishing a common foundation and a shared understanding of interprofessional collaboration through early IPE activities can serve as a springboard for subsequent learning activities, supporting progressive development throughout the educational journey. Reflective writing has previously proven to be a powerful tool in the development of interprofessional competence (e.g. [5, 45]), and the current study serves as another example of this. The value of reflective writing can be further enhanced if the reflections become part of a larger portfolio encompassing several interprofessional activities [44].

In summary, this study supports the early curricular introduction of IPE. Following the completion of the first-year IPE module, students shared wide-ranging reflections on interprofessional collaboration, which provided valuable insight into their understanding of interprofessional communication and trust, shared knowledge and organisational factors that impact on collaboration.

## Conclusions

This study contributes to the discussion concerning the value of introducing IPE at an early stage of health professions education. Despite being in such an early stage of their education, students demonstrated a rich understanding of the purpose of interprofessional collaboration, both in enhancing the quality of patient care and in contributing to their effectiveness as future members of interprofessional teams for sustainable working life.

Thus, even without direct team-based or clinical experience, students can still gain insights into, for example, the consequences of hierarchies and power dynamics for a holistic approach to patient care—insights that could be expected to emerge through practical experience in clinical settings. This awareness can be fostered through IPE activities designed to address interprofessional aspects in small-group settings with students from other professions.

Understanding the students’ novel perspectives of interprofessional collaboration provides insights that can inform the development of educational strategies, enabling educators to design relevant learning activities that support a progression in students’ ongoing education. Finally, we demonstrated that incorporating brief reflective writings into an IPE module helps students internalise and understand interprofessional competencies as part of their learning process which is of relevance for future educational development as well as research, as it emphasises the value of reflection as a means of capturing students’ perceptions.

## Data Availability

The data used and analysed during the current study are available from the corresponding author on reasonable request.

## Abbreviations

BMLS: Biomedical Laboratory Science Programme
ECTS: European Credit Transfer System
IPTW: Interprofessional training ward
IPEC: Interprofessional Education Collaborative
IPE: Interprofessional education
IPCP: Interprofessional collaborative practice
M: Medical Programme
N: Nursing Programme
OT: Occupational Therapy Programme
PBL: Problem-based learning
PT: Physiotherapy Programme
SLP: Speech and Language Pathology Programme

## References

[1] Bloomfield J, van Diggele C, Frotjold A, Schneider C, Howard R, Roberts C, Lane S. Achieving the unachievable: the development of a large-scale interprofessional education workshop for first-year health professional students. Developing a large-scale interprofessional education workshop. Med Res Arch. 2024;12(3).

[2] Hanson C, Custer T, Schmidt C, Hartman T, Lyden E, List S, Wampler K, Michael K. Following the growth of sarah’s baby: an interprofessional education activity for medical nutrition education and diagnostic medical sonography students. J Interprof Educ Pract. 2017;7:17–20.

[3] Lee CA, Pais K, Kelling S, Anderson OS. A scoping review to understand simulation used in interprofessional education. J Interprof Educ Pract. 2018;13:15–23.

[4] Lunde L, Moen A, Jakobsen RB, Rosvold EO, Braend AM. Exploring healthcare students’ interprofessional teamwork in primary care simulation scenarios: collaboration to create a shared treatment plan. BMC Med Educ. 2021;21(1):416. 34344334 10.1186/s12909-021-02852-z PMC8336096

[5] Edelbring S, Broberger E, Sandelius S, Norberg J, Wiegleb Edström D. Flexible interprofessional student encounters based on virtual patients: a contribution to an interprofessional strategy. J Interprof Care. 2022;36(2):310–7. 33955312 10.1080/13561820.2021.1893287

[6] Anderson ES, Ford J, Kinnair DJ. Interprofessional education and practice guide 6: developing practice-based interprofessional learning using a short placement model. J Interprof Care. 2016;30(4):433–40. 27269042 10.3109/13561820.2016.1160040

[7] Bivall A-C, Lindh Falk A, Gustavsson M. Students’ interprofessional workplace learning in clinical placement. Professions Professionalism. 2021;11(3).

[8] Brack P, Shields N. Short duration clinically-based interprofessional shadowing and patient review activities May have a role in Preparing health professional students to practice collaboratively: a systematic literature review. J Interprof Care. 2018;33:1–10. 30395747 10.1080/13561820.2018.1543256

[9] Fenn N, Mushkat Z, Murray AN, Dimalanta K, Vandiver M, Robbins ML, Hulme J, Dupre AM. Interprofessional education for complex neurological cases. J Interprof Care. 2020;34(6):784–90. 31851543 10.1080/13561820.2019.1691159

[10] Naumann F, Schumacher U, Stuckey A, Love A, Thompson C, Tunny R, Nash R. Developing the next generation of healthcare professionals: the impact of an interprofessional education placement model. J Interprof Care. 2021;35(6):963–6. 33784925 10.1080/13561820.2021.1879749

[11] Törnqvist T, Lindh Falk A, Tingström P. Sharing knowledge: Final-year healthcare students working together at an interprofessional training ward. J Interprof Educ Pract. 2023;33:100670.

[12] Mink J, Mitzkat A, Krug K, Mihaljevic A, Trierweiler-Hauke B, Götsch B, Wensing M, Mahler C. Impact of an interprofessional training ward on interprofessional competencies – a quantitative longitudinal study. J Interprof Care. 2020;35:1–9. 32841067 10.1080/13561820.2020.1802240

[13] Zelic L, Bolander Laksov K, Samnegard E, Ivarson J, Sondén A. Call the on-Call: authentic team training on an interprofessional training Ward – A case study. Adv Med Educ Pract. 2023;14:875–87. 37588849 10.2147/AMEP.S413723 PMC10426451

[14] Karlsson EA, Kvarnström S, Kvarnström M. Exploring a revised interprofessional learning curriculum in undergraduate health education programs at Linköping university. BMC Med Educ. 2024;24(1):466. 38671441 10.1186/s12909-024-05458-3 PMC11055219

[15] Khalili H, Orchard C, Laschinger HK, Farah R. An interprofessional socialization framework for developing an interprofessional identity among health professions students. J Interprof Care. 2013;27(6):448–53. 23777592 10.3109/13561820.2013.804042

[16] O’Keefe M, Henderson A, Chick R. Defining a set of common interprofessional learning competencies for health profession students. Med Teach. 2017;39(5):463–8. 28332419 10.1080/0142159X.2017.1300246

[17] Berger-Estilita J, Fuchs A, Hahn M, Chiang H, Greif R. Attitudes towards interprofessional education in the medical curriculum: a systematic review of the literature. BMC Med Educ. 2020;20(1):254. 32762740 10.1186/s12909-020-02176-4 PMC7410157

[18] Olson R, Bialocerkowski A. Interprofessional education in allied health: a systematic review. Med Educ. 2014;48(3):236–46. 24528458 10.1111/medu.12290

[19] Gunaldo T, Augustus-Wallace A, Brisolara K, Norman M, Mercante D, Synco T, Zorek J, Schilling D. Improving stereotypes: the impact of interprofessional education in pre-health students. J Interprof Care. 2021;35:1–5. 10.1080/13561820.2020.1806218 32838602

[20] Coster S, Norman I, Murrells T, Kitchen S, Meerabeau E, Sooboodoo E, d’Avray L. Interprofessional attitudes amongst undergraduate students in the health professions: a longitudinal questionnaire survey. Int J Nurs Stud. 2008;45(11):1667–81. 18423644 10.1016/j.ijnurstu.2008.02.008

[21] Hoffman S, Harnish D. The merit of mandatory interprofessional education for pre-health professional students. Med Teach. 2007;29:e235–242. 18236267 10.1080/01421590701551672

[22] Reeves S, Fletcher S, Barr H, Birch I, Boet S, Davies N, McFadyen A, Rivera J, Kitto S. A BEME systematic review of the effects of interprofessional education: BEME guide 39. Med Teach. 2016;38(7):656–68. 27146438 10.3109/0142159X.2016.1173663

[23] Lockeman KS, Lanning SK, Dow AW, Zorek JA, DiazGranados D, Ivey CK, Soper S. Outcomes of introducing early learners to interprofessional competencies in a classroom setting. Teach Learn Med. 2017;29(4):433–43. 28281832 10.1080/10401334.2017.1296361

[24] Lieberman-Betz RG, Brown JA, Wiegand SD, Vail CO, Fiss AL, Carpenter LJ. Building collaborative capacity in early intervention preservice providers through interprofessional education. Lang Speech Hear Serv Sch. 2023;54(2):504–17. 36749761 10.1044/2022_LSHSS-22-00110

[25] Kemp S, Brewer M. Early stages of learning in interprofessional education: stepping towards collective competence for healthcare teams. BMC Med Educ. 2023;23(1):694. 37740200 10.1186/s12909-023-04665-8 PMC10517498

[26] Interprofessional Education Collaborative: Core Competencies for Interprofessional Collaborative Practice. Version 3. Washington, D.C. 2023. https://www.ipecollaborative.org/core-competencies.

[27] Canadian Interprofessional Health Collaborative. CIHC competency framework for advancing collaboration 2024. Ottawa; 2024. https://cihc-cpis.com/.

[28] Imafuku R, Kataoka R, Ogura H, Suzuki H, Enokida M, Osakabe K. What did first-year students experience during their interprofessional education? A qualitative analysis of e-portfolios. J Interprof Care. 2018;32(3):358–66. 29364744 10.1080/13561820.2018.1427051

[29] Peeters M, Sexton M. Use of reflective writing within interprofessional education: a mixed-methods analysis. J Interprof Care. 2020;34:307–14. 31525128 10.1080/13561820.2019.1649644

[30] Areskog N-H. Multiprofessional education at the undergraduate level – the Linköping model. J Interprof Care. 1994;8(3):279–82.

[31] Wilhelmsson M, Pelling S, Ludvigsson J, Hammar M, Dahlgren LO, Faresjö T. Twenty years experiences of interprofessional education in Linköping – ground-breaking and sustainable. J Interprof Care. 2009;23(2):121–33. 19225972 10.1080/13561820902728984

[32] Lindh Falk A, Dahlberg J, Ekstedt M, Heslyk A, Whiss P, Abrandt Dahlgren M. Creating spaces for interprofessional learning: strategic revision of a common IPL curriculum in undergraduate programmes. In: Interprofessional education in Europe: policy and practice. edn. Edited by Vyt A, Pahor M, Tervaskanto-Maentausta T. Antwerp: Garant Publishers Limited; 2015: 49–66.

[33] Barr H, Ford J, Gray R, Helme M, Hutchings M, Low H, Machin A, Reeves S. Interprofessional education guidelines. Centre for the Advancement of Interprofessional Education. Fareham. 2017. https://www.caipe.org/resources/caipe-2017-interprofessional-education-guidelines-barr-h-ford-j-gray-r-helme-m-hutchings-m-low-h-machin-a-reeves-s/

[34] Imafuku R, Kataoka R, Mayahara M, Suzuki H, Saiki T. Students’ experiences in interdisciplinary Problem-based learning: A discourse analysis of group interaction. Interdiscip J Probl Based Learn. 2014;8(2).

[35] Dahlgren LO. Interprofessional and problem-based learning: a marriage made in heaven? J Interprof Care. 2009;23(5):448–54. 20602585 10.1080/13561820903163579

[36] Braun V, Clarke V. Thematic analysis: A practical guide. Thousand Oaks: SAGE; 2021.

[37] Alfasoft NVivo. 2024. https://alfasoft.com/uk/software/statistics-and-data-analysis/qda-qualitative-data-analysis/nvivo/.

[38] Braun V, Clarke V. Successful qualitative research: A practical guide for beginners. First edn: Sage; 2013.

[39] Barr H. Interprofessional education: Today, yesterday and tomorrow. A review. The learning and teaching support network for health sciences & practice. The UK Centre for the Advancement of Interprofessional Education (CAIPE); London. 2002.

[40] Törnqvist T, Ekstedt M, Wiggins S, Abrandt Dahlgren M. Connecting knowledge: First-year health care students’ learning in early interprofessional tutorials. J Interprof Care. 2023;37(5):758–66. 36588170 10.1080/13561820.2022.2162021

[41] Jentoft R. Boundary-crossings among health students in interprofessional geropsychiatric outpatient practice: collaboration with elderly people living at home. J Interprof Care. 2020;35:1–10. 32233885 10.1080/13561820.2020.1733501

[42] World Health Organization: Framework for action on interprofessional education & collaborative practice. Geneva. 2010. https://www.who.int/publications/i/item/framework-for-action-on-interprofessional-education-collaborative-practice. 21174039

[43] Gunaldo TP, Baker-Rush ML, Smeets HWH, Xyrichis A. Rethinking interprofessional language: a call for a living definition. J Interprof Care. 2025;39(6):1–5. 10.1080/13561820.2025.2480557 40166952

[44] Domac S, Anderson ES, Smith R. Learning to be interprofessional through the use of reflective portfolios? Soc Work Educ. 2016;35(5):530–46.

[45] Roy V, Collins L, Sokas C, Lim E, Umland E, Speakman E, Koeuth S, Jerpbak C. Student reflections on interprofessional education: moving from concepts to collaboration. J Allied Health. 2016;45:109–12. 27262468

[46] World Medical Association. WMA Declaration of Helsinki – Ethical principles for medical research involving human participants [cited Oct 24 2025]; Available from: https://www.wma.net/what-we-do/medical-ethics/declaration-of-helsinki. 10.1001/jama.2024.21972 39425955

